# Are health care inequalities unfair? A study on public attitudes in 23 countries

**DOI:** 10.1186/s12939-016-0350-8

**Published:** 2016-04-11

**Authors:** Olaf von dem Knesebeck, Nico Vonneilich, Tae Jun Kim

**Affiliations:** Department of Medical Sociology, University Medical Center Hamburg-Eppendorf, Martinistr. 52, 20246 Hamburg, Germany

**Keywords:** Health care inequalities, Public attitudes, Perception of unfairness, International comparison, International Social Survey Programme, Welfare states

## Abstract

**Background:**

In this article we focus on the following aims: (1) to analyze national and welfare state variations in the public perception of income-related health care inequalities, (2) to analyze associations of sociodemographic, socioeconomic, health-related, and health care factors with the perception of health care inequalities.

**Methods:**

Data were taken from the International Social Survey Programme (ISSP), an annually repeated cross-sectional survey based on nationally representative samples. 23 countries (*N* = 37,228) were included and assigned to six welfare states. Attitude towards income-related health care inequalities was assessed by asking: “Is it fair or unfair that people with higher incomes can afford better health care than people with lower incomes?” with response categories ranging from “very fair” (1) to “very unfair” (5). On the individual level, sociodemographic (gender, age), socioeconomic (income, education) health-related (self-rated health), and health care factors (health insurance coverage, financial barriers to health care) were introduced.

**Results:**

About two-thirds of the respondents in all countries think that it is unfair when people with higher incomes can afford better health care than people with lower incomes. Percentages vary between 42.8 in Taiwan and 84 in Slovenia. In terms of welfare states, this proportion is higher in Conservative, South European, and East European regimes than in East Asian, Liberal, and Social-Democratic regimes. Multilevel logistic regression analyses show that women, people affected by a low socioeconomic status, poor health, insufficient insurance coverage, and foregone care are more likely to perceive income-related health care inequalities as unfair.

**Conclusions:**

In most countries a majority of the population perceives income-related health care inequalities as unfair. Large differences between countries were observed. Welfare regime classification is important for explaining the variation across countries.

## Background

The provision of equal access to health care is a core objective of many health care systems. Social inequalities in access to health services evolve if persons with a higher socioeconomic status (SES) are more likely to receive medical care, compared to those with a lower SES when the same need is given [1, 2]. Especially income was found to constitute a major determinant of access to health services, since it is associated with the risk to delay or forgo needed medical care [3–5]. People with a low income generally tend to have more difficulties in accessing medical care and are more likely to experience unmet medical needs due to financial reasons. Although this association was found in a number of different countries [3–9], cross-national differences in the magnitude are evident.

In terms of international comparisons, the importance of welfare state arrangements as a determinant of health and health care was highlighted in a number of studies [10–13]. Originating from Esping-Andersen’s [14] three-fold typology, welfare regimes were grouped upon their overall provision of social protection by explicitly referring to the three dimensions of decommodification, social stratification as well as the role of the family and the state in the provision of welfare. As such, Esping Andersen characterized the social democratic regime as universalistic, exemplified by a strong redistributive social security system with relatively generous social transfers and social protection. In the Conservative (or Bismarckian) regime, the supply of benefits is often earnings orientated, while the family is stressed as a main source of social protection. The liberal regime is distinguished by its minimal provision of welfare, modest social transfers and an emphasis of the market. Ever since, a growing body of literature attempted to further elaborate and extend Esping-Andersen’s typology. As such, scholars suggested Israel as a Liberal regime due to its limited social insurance [15, 16], while others included Southern (characterized by fragmented welfare provision and partial coverage of health services) and Eastern European (characterized by limited health service provision and an accentuation of marketization as well as decentralization) welfare regimes [12, 17, 18]. In addition, researchers specifically discussed the applicability of welfare regimes typologies to non-European countries. In this regard, a Confucian or East Asian welfare state was introduced that is characterized by low to medium social security expenditure, high family welfare responsibility and low levels of government intervention [19, 20].

Although income-related health care inequalities seem to be a global phenomenon [3–9] and are an important political and public health issue, there is not much known about the public attitudes towards such inequalities [21, 22]. If people perceive health care inequalities as unacceptable or unfair, this can have a negative impact on their assessment of and trust in the health care system [22]. There is evidence that the level of trust in the health care system is a crucial factor for the help-seeking behavior, utilization of services, the relationship between patient and provider, and patient compliance [23, 24]. In other words, perceptions of health care inequalities lead to decreased levels of trust in the health care system, which, in turn, can be expected to negatively affect utilization and quality of health care.

Against this background, in this article we focus on the following aims: (1) To analyze national and welfare state variations in the public perception of income-related health care inequalities, (2) to analyze associations of sociodemographic, socioeconomic, health-related, and health care factors with perceptions of health care inequalities.

## Methods

### Data

Data were taken from the International Social Survey Programme (ISSP). The ISSP is an annually repeated cross-sectional survey based on nationally representative samples, covering a variety of sociological topics since 1985 (e.g. religion, national identity, social inequality or environment). The present analysis is based on the module “health and health care” including 32 countries and a total of 55,081 participants. The module deals with health related issues, such as self-reported health and well-being, personal experiences with health care (barriers to treatment and regular care due to inability to pay, distance from services, no familiarity with medical system, etc.), confidence and trust in the health care system, relative importance placed on various health care fields, health care regimes, and satisfaction with health care services. We excluded nine countries (Bulgaria, Croatia, Lithuania, Russia, Chile, China, Philippines, South Africa, and Turkey) because they have to be considered in a state of transition where a definite social security system has not been established yet and the literature on welfare state typology is not clear on how to classify these countries [16, 17]. Based on previous studies and the considerations outlined in the Introduction, the remaining 23 countries (*N* = 37,228) were assigned to six welfare states (Table 1): Liberal (Australia, Great Britain, Israel, United States), Conservative (Belgium, France, Germany, Netherlands, Switzerland), Social-Democratic (Denmark, Finland, Norway, Sweden), South European (Italy, Portugal, Spain), East Asian (Japan, South Korea, Taiwan), and East European (Czech Republic, Poland, Slovak Republic, Slovenia).

**Table 1:** Sample Characteristics of the International Social Survey Programme (ISSP) 2011 (23 countries, *N* = 37,228)

Country/welfare state	N	Response rate (%)	Age (mean)	Sex (female, %)	Perception of health care inequalities (unfair, %)
Australia	1946	31.1	55.1	52.8	50.4
Great Britain	936	53.9	49.7	56.7	46.4
Israel	1220	66.7	45.8	55.8	70.7
United States	1550	78.2	50.0	56.7	56.2
Liberal	5652	57.5	50.7	55.1	55.7
Belgium	3083	35.8	49.7	53.8	75.8
France	3319	35.9	52.1	58.4	80.8
Germany	1681	37.7	50.0	49.3	77.7
Netherlands	1472	33.7	54.0	55.5	79.9
Switzerland	1212	53.9	48.9	49.2	66.8
Conservative	10,767	39.4	50.9	54.2	77.2
Denmark	1388	56.1	46.3	50.4	61.5
Finland	1340	53.7	46.2	55.1	45.3
Norway	1834	48.5	48.3	53.4	72.5
Sweden	1158	59.8	50.0	52.6	76.0
Social-Democratic	5720	54.5	47.7	52.9	64.2
Italy	1186	23.0	50.7	53.7	79.4
Portugal	1022	58.6	51.6	58.2	74.4
Spain	2712	67.8	49.2	51.8	73.9
South European	4920	49.8	50.1	53.6	75.2
Japan	1306	73.9	50.5	52.8	62.1
Korea (South)	1535	61.4	46.0	55.1	46.8
Taiwan	2199	50.1	46.8	50.6	42.8
East Asian	5040	61.8	47.5	52.6	49.0
Czech Rep.	1804	57.9	47.4	55.3	71.0
Poland	1115	42.6	47.8	54.0	73.5
Slovak Rep.	1128	47.1	51.9	53.6	72.0
Slovenia	1082	64.7	48.6	54.5	84.0
East European	5129	53.1	48.8	54.5	74.5
*Total*	*37,228*	*51.8*	*49.5*	*53.9*	*67.4*

In most countries, fieldwork was initiated in 2011 and the data collection for the included 23 countries was completed in 2013. Statistical data and a comprehensive documentation are freely available at the Leibniz Institute for the Social Sciences webpage (http://www.gesis.org/issp). Sample sizes varied from 936 in Great Britain to 3319 in France. For most countries, respondents were aged 18 years or older, except for Finland, Italy, Japan, Norway, and Sweden, where respondents were 16 or older. Sampling procedures and modes of data collection varied between countries. The selection method of participants differed from simple to multi-stage stratified random samples. Samples were designed to be representative for the adult population in the respective country. For the collection of data, face-to-face, paper and pencil interviews (PAPI) or computer assisted personal interviews (CAPI) with standardized questionnaires were used (Czech Republic, Germany, Great Britain, Israel, Japan, South Korea, Poland, Portugal, Slovenia, Spain, Slovak Republic, Switzerland, Taiwan, USA). Other countries referred to a postal self-completion (Australia, France, Italy, Netherlands, Sweden) or web-based questionnaires (Denmark). As for the remaining countries, mixed modes for the assessment were considered (Belgium, Finland, Norway). Country-specific response rates ranged from 23 % in Italy to 78.2 % in the U.S. (Table 1). Informed consent was considered to have been given when individuals completed the interview. According to the International Survey Programme ethical statement (http://www.issp.org), all ISSP members must comply with the given legal requirements in each country. Before depositing data to the ISSP Archive, national ISSP data are anonymized so that individual survey participants cannot be identified. Given these regulations, no further ethical approval for the specific analyses presented here was needed.

### Measures

The attitude towards income-related health care inequalities was assessed by the following question: “Is it fair or unfair that people with higher incomes can afford better health care than people with lower incomes?” (response categories: “very fair (1)”, “somewhat fair (2)”, neither fair nor unfair (3)”, “somewhat unfair (4)” and “very unfair (5)”). For the analyses, the variable was dichotomized by combining the first three (fair/neither…nor) and the last two categories (unfair).

Sociodemographic (gender, age), socioeconomic (income, education) health-related (self-rated health), and health care factors (health insurance coverage, financial barriers to health care) were introduced as predictors (Tables 1 and 2).

**Table 2:** Distribution of the individual level factors (International Social Survey Programme (ISSP) 2011 (23 countries, *N* = 37,228))

Country/welfare state	Education (lower sec. school or less, %)	Income (lowest tertile, in US$, per month)	Self-rated health (fair/poor, %)	Health insurance coverage (not well covered, %)	Forgone care (yes, %)
Australia	28.7	2354	18.7	19.3	9.2
Great Britain	49.4	1474	25.8	1.6	5.6
Israel	36.5	870	18.3	15.7	8.9
United States	11.4	1500	23.7	12.3	12.1
Liberal	28.7	1481	21.2	13.5	9.6
Belgium	32.9	1656	28.7	5.7	11.5
France	48.2	1820	20.6	12.3	7.8
Germany	11.7	1517	27.2	3.6	4.7
Netherlands	48.1	1540	26.2	4.1	2.8
Switzerland	19.7	4366	10.1	3.5	1.9
Conservative	34.8	1750	23.5	6.9	7.0
Denmark	9.1	3375	21.3	6.8	9.8
Finland	19.2	2800	26.8	20.1	10.1
Norway	28.3	4800	27.1	10.2	4.4
Sweden	42.3	2667	11.0	13.0	3.7
Social-Democratic	23.9	3375	22.4	13.1	6.9
Italy	36.8	1307	39.0	23.8	7.3
Portugal	64.1	617	49.7	27.8	11.0
Spain	53.3	840	23.6	5.3	3.4
South European	51.6	840	32.7	14.0	5.9
Japan	21.0	1800	28.6	10.9	3.8
Korea (South)	23.1	1179	19.8	11.5	6.3
Taiwan	33.4	612	53.5	33.5	1.8
East Asian	27.1	988	32.3	21.2	3.5
Czech Rep.	39.1	784	24.6	17.5	2.4
Poland	20.5	350	37.6	52.4	14.6
Slovak Rep.	45.7	525	26.6	21.0	4.0
Slovenia	39.1	840	30.7	13.5	1.3
East European	36.5	588	29.2	24.9	4.7
*Total*	*33.7*	*1284*	*26.0*	*14.1*	*6.5*

Information on disposable income and size of household were summarized according to the ‘OECD-modified scale’ [25] to calculate the monthly net household equivalent income. The respondent was attributed with a weight of 1, while every additional household member was given a weight of 0.5. Net household equivalent income was converted into US$ using the average exchange rates in the year 2011 and divided into country specific tertiles. Education was measured according to the International Standard Classification of Education (ISCED) [26]. “No formal education”, “Primary school” and “Lower secondary school” represented a low, while “Upper secondary (allowing entry to university)” and “Post-secondary and non-tertiary” were coded as medium educational level. “Lower level tertiary (also technical schools)” and “Upper level tertiary” were coded as high educational level. For the assessment of health, respondents were asked to rate their general subjective health on a 5-point Likert scale (“excellent”, “very good”, “good”, “fair”, and “poor”). Health insurance status was ascertained by asking respondents: “Thinking about your health insurance coverage would you say you are (1) well covered or (2) not well covered?” Financial barriers to health care were assessed by asking: “During the past 12 months did it ever happen that you did not get medical treatment you needed because you could not pay for it?” (yes/no). In line with other studies we label this as ‘forgone care’ [8, 27, 28].

### Analyses

To analyze national and welfare state variations in the public perception of income-related health care inequalities, descriptive statistics were used. Multilevel logistic regression techniques were utilized to analyze associations. First, an empty model (model 0) was calculated to analyze the variance in the public perception attributed to country differences. In model 1, the individual level indicators (gender, age, income, education, self-rated health, health insurance coverage, and foregone care) were introduced. Model 2 additionally included the six welfare regimes, with the Social Democratic regime as the reference category. Odds ratios, 95 %-confidence intervals, significances, the intra-class correlation coefficient (ICC), based on the between-country variance, and deviance of the statistical models are documented. All statistical analyses were conducted using the software *R* (Version 3.2.1) and *RStudio* (Version 0.99.447), including the R packages *lme4, lmerTest, lattice, sjPlot, nlme, car, digest, ggplot2* and *haven*.

## Results

Table 1 (last column) shows that about two-thirds of the respondents (67.4 %) in all countries think that it is unfair when people with higher incomes can afford better health care than people with lower incomes. Percentages vary between 42.8 in Taiwan and 84 in Slovenia. In terms of welfare states, this proportion is higher in Conservative, South European, and East European regimes than in East Asian, Liberal, and Social-Democratic regimes.

Table 3 shows the results of the multi-level analyses. In Model 0, the empty model, the ICC of 0.094 indicates that about 9 % of the variation in the perceived unfairness of health care inequalities can be explained by differences between countries. In the next step (Model 1), the individual level variables were introduced. People in the medium and low income tertiles have a significantly increased likelihood of perceiving inequalities in health care as unfair, compared to respondents in the high income tertile. Moreover, women, respondents with low education, and worse than very good health as well as people who experienced insufficient health insurance coverage and forgone care show increased likelihoods of perceiving health care inequalities as unfair. After adjustment of the individual level characteristics, about 10 % of the perceived unfairness of health care inequalities is due to differences between countries. In Model 2, the six welfare regimes were introduced. Compared to respondents living in the Social-Democratic regime, those in Conservative regimes are significantly more likely to perceive health care inequalities as unfair. The opposite is true for people living in an East Asian welfare regime, as they are significantly less likely to perceive health care inequalities as unfair. After introduction of the welfare regimes, the variation in perceived fairness that can be attributed to differences between countries is reduced to 3.8 %.

**Table 3:** Multilevel models for perceived unfairness of health care inequalities (International Social Survey Programme (ISSP) 2011 (N_indviduals_ = 37,228))

	Model 0			Model 1			Model 2		
	OR	CI	*p*	OR	CI	*p*	OR	CI	*p*
Fixed Parts									
(Intercept)	2.11	1.66–2.69	**<0.001**	1.16	0.89–1.50	0.268	0.99	0.69–1.43	0.976
Equivalent household income (0 = highest tertile)									
Medium				1.31	1.22–1.40	**<0.001**	1.31	1.22–1.40	**<0.001**
Low				1.46	1.35–1.57	**<0.001**	1.46	1.35–1.57	**<0.001**
Educational status (0 = lower level tertiary or higher)									
Medium				1.05	0.98–1.13	0.180	1.05	0.98–1.13	0.199
Low				1.12	1.03–1.22	**0.007**	1.12	1.03–1.21	**0.007**
Age (0 = < 40 years)									
40–60 years				1.02	0.95–1.09	0.594	1.02	0.95–1.09	0.599
>60 years				0.93	0.86–1.01	0.071	0.93	0.86–1.01	0.072
Sex (0 = male)				1.49	1.41–1.58	**<0.001**	1.49	1.41–1.58	**<0.001**
Subjective health (0 = excellent/very good)									
Good				1.08	1.01–1.16	**0.019**	1.08	1.01–1.16	**0.020**
Fair/poor				1.33	1.23–1.45	**<0.001**	1.33	1.23–1.45	**<0.001**
Insurance coverage (0 = well covered)				1.31	1.20–1.44	**<0.001**	1.31	1.19–1.44	**<0.001**
Forgone care (0 = no)				1.24	1.06–1.44	**0.007**	1.24	1.06–1.44	**0.007**
Welfare regimes (0 = Social-Democratic)									
Conservative							1.89	1.17–3.07	**0.010**
Liberal							0.72	0.43–1.21	0.214
South European							1.71	0.99–2.98	0.056
East European							1.65	0.98–2.75	0.058
East Asian							0.51	0.29–0.89	**0.017**
Random Parts									
ICC_country_	0.094			0.098			0.038		
Between-country variation	0.345			0.357			0.131		
Deviance	28,967			28,413			28,390		
N_country_	23			23			23		

*Abbreviations*: *OR* odds ratios, *CI* confidence intervals, *p* significances, *ICC* intra-class correlation coefficients, *AIC* Akaike information criterion, *N* number of cases

Significant associations (p<0.05) are bold

## Discussion

In this study, national and welfare state variations in the public perception of income-related health care inequalities were analyzed in 23 countries based on the International Social Survey Programme (ISSP). Moreover, associations of sociodemographic, socioeconomic, health-related, and health care factors with perceptions of health care inequalities were examined. Results show that about two thirds of the people in all countries think that it is unfair when people with higher incomes can afford better health care than people with lower incomes. However, large differences between countries and welfare states were observed. Perception of unfairness is least pronounced (below 50 %) in the East Asian welfare state, while we found frequencies of more than 70 % in Conservative as well as in South and East European regimes. As this is the first study on national and welfare state variations in the public perception of health care inequalities, the analyses were not driven by specific hypotheses. However, findings of the multilevel analyses indicate that the welfare regime classification is important for explaining the variation in attitudes towards health care inequalities across countries.

As Dahl and van der Wel [11] have pointed out, welfare resources produce human capital, promote human agency, and provide capacities to cope with stressful events as well as remove exposures to health risks. And while increased welfare provisions might aid in securing a social climate of civicness [16], according to regime theory [29], welfare arrangements (culturally integrated in the welfare institution) provide generalized frames that individuals can refer to as normal or appropriate. Following these assumptions of a regime theory, lower degrees of perceived unfairness are to be expected in regimes with higher distributive policies. However, results revealed that perception of unfairness is not least pronounced in the Social-Democratic welfare state which is defined by a universalistic approach to social rights [30], a high degree of decommodification and an increased distributive policy [29]. As for the liberal welfare regime, the rather low proportion of people perceiving health care inequalities as unfair correspond with the limited welfare model in which social and health insurance mechanisms are market-dependent while public social expenditures are reserved only for the needy [30, 31]. Redistributive policies in the Conservative welfare state are considered weaker than in the Social-Democratic, yet stronger than the liberal welfare regime [31]. Status differentiating welfare programs in which benefits are often earnings related and geared towards maintaining existing social patterns as well as the focus on the family as a unit of benefit may help to understand the comparatively high proportion of people perceiving health care inequalities as unfair in the Conservative welfare state regime [30]. A high level of perceived unfairness was also observed in the Southern welfare state. Some authors refer to the Latin Rim as a residual subcluster of the Conservative welfare regime [14], since Southern welfare regimes are characterized by high fragmented income maintenance system with limited welfare provision [32, 33], in which the provision of health care is connected to employment and the family [18, 34]. As for the East European welfare type, also high proportions of perceived unfairness towards health care inequalities were evident. However, due to the still undergoing extensive social reforms towards marketization, the decentralization of health insurance [35] and limited health service provision [32], an interpretation of the results according to the regime theory is premature. Finally, the lowest frequencies of labeling health care inequalities as unfair were found in the East Asian (or Confucian) welfare state, which is characterized by low to medium social security expenditure, underdeveloped public service provision and low government regulation [19, 20]. Even though the family is assigned a major responsibility in the provision of welfare (similar to the Conservative and South European welfare regimes), the rather low levels of unfairness perceptions might be affected by the emphasis on social ethics (e.g. thrift, diligence, and work ethic) that reflect economies derived from Confucianism [32].

Furthermore, our results show that women, people affected by a low socioeconomic status, poor health, insufficient insurance coverage, and foregone care are more likely to perceive income-related health care inequalities as unfair. However, these individual level characteristics do not seem to explain much of the country variations in the public attitudes towards health care inequalities. After introduction of the welfare states, associations of the individual level factors with the perception of health care inequalities hardly change. Thus, the effect of the sociodemographic, socioeconomic, health-related, and health care factors is not mediated by factors characterizing the countries.

Several methodological aspects should be considered when interpreting our findings. Although the ISSP is an international survey that strives for high methodological standards (http://www.gesis.org/issp), sampling procedures and modes of data collection varied between countries potentially limiting the comparability of the data (see Methods). Moreover, there are large variations in the response rates (23 % in Italy, 78.2 % in the U.S.). Response rates are lower than 50 % in nine of the 23 countries (Austria, Belgium, France, Germany, Netherlands, Norway, Italy, Poland, and Slovak Republic). External validity is threatened as we cannot rule out a selection bias due to non-response. If our estimates are sensitive to response rates, the comparability of the estimates for different countries would be reduced. Results from survey research indicate that response rates are lower in lower socioeconomic groups and in less healthy people [36]. This could imply that non-response might lead to an underestimation of the associations analysed here. In terms of the question measuring the attitude towards income-related health care inequalities (“Is it fair or unfair that people with higher incomes can afford better health care than people with lower incomes?”), applicability in cross-national studies has not yet been established. It has to be taken into account that respondents from different countries and cultures will have different reference levels and notions of unfairness and good health care. Finally, as we dichotomized the dependent variable for the multilevel logistic regression analyses, results on associations to some extent are crude. Nevertheless, we decided to dichotomize the variable and use logistic regression models for the sake of clearness, and due to the distribution characteristics of the dependent variable. Additional analyses using multilevel linear regression techniques and continuous variables (not shown) revealed that results essentially remain stable.

## Conclusions

Despite these limitations, our results show that a majority of the population perceives income-related health care inequalities as unfair in most countries and welfare states, and that the welfare regime classification is important for explaining the variation in attitudes towards health care inequalities across countries. Perceptions of unfairness can have a negative impact on trust in the health care system and on the utilization and quality of health care [22–24]. Our findings indicate that such perceptions of unfairness are particularly pronounced among deprived people with poor health. If these people thereby lose trust in the health care system, this may further increase inequalities in the utilization and quality of health care.
